# New Organic–Inorganic Salt Based on Fluconazole Drug: TD-DFT Benchmark and Computational Insights into Halogen Substitution

**DOI:** 10.3390/ijms23158765

**Published:** 2022-08-06

**Authors:** Hela Ferjani, Rim Bechaieb, Marzook Alshammari, O. M. Lemine, Necmi Dege

**Affiliations:** 1 Chemistry Department, College of Science, Imam Mohammad Ibn Saud Islamic University (IMISU), Riyadh 11623, Saudi Arabia; 2 Selective Organic & Heterocyclic Synthesis-Biological Activity Evaluation, Département de Chimie, Faculté des sciences de Tunis, Université de Tunis El Manar, Tunis 2092, Tunisia; 3 Laboratoire de Chimie Théorique, Sorbonne Universités, UPMC Univ Paris 06, UMR 7616, F-75005 Paris, France; 4 The National Center for Nanotechnology and Semiconductors, KACST, Riyadh 11442, Saudi Arabia; 5 Department of Physics, College of Sciences, Imam Mohammad Ibn Saud Islamic University (IMISU), Riyadh 11623, Saudi Arabia; 6 Department of Physics, Faculty of Arts and Sciences, Ondokuz Mayıs University, 55139 Samsun, Turkey

**Keywords:** crystal structure, fluconazole drug, salt, spectroscopy, optical and magnetic studies, DFT, TD-DFT, chemical reactivity descriptors, ICT

## Abstract

In this study, we report the synthesis of a new organic–inorganic molecular salt of the clinically used antifungal drug fluconazole, (H2Fluconazole).SnCl_6_.2H_2_O. By detailed investigation and analysis of its structural properties, we show that the structure represents a 0D structure built of alternating organic and inorganic zig-zag layers along the crystallographic c-axis and the primary supramolecular synthons in this salt are hydrogen bonding, F···π and halogen bonding interactions. Magnetic measurements reveal the co-existence of weak ferromagnetic behavior at low magnetic field and large diamagnetic contributions, indicating that the synthesized material behaves mainly as a diamagnetic material, with very low magnetic susceptibility and with a band gap energy of 3.6 eV, and the salt is suitable for semiconducting applications. Extensive theoretical study is performed to explain the acceptor donor reactivity of this compound and to predict the Cl-substitution effect by F, Br and I. The energy gap, frontier molecular orbitals (FMOs) and the different chemical reactivity descriptors were evaluated at a high theoretical level. Calculations show that Cl substitution by Br and I generates compounds with more important antioxidant ability and the intramolecular charge transfer linked to the inorganic anion.

## 1. Introduction

The great technological potential of hybrid organic–inorganic materials as low-cost solar-cell absorbers, phosphors, and light-emitting diodes [1,2,3,4] has renewed interest in this large and well-known family of materials. The synthetic versatility and structural definition of these crystalline solids, which are typically formed through solution-state self-assembly, drive research in this field. Many possible combinations of organic amine (A), metal (B = Sn or Pb) and halides (X) within the A_a_B_b_X_3b+a_ hybrid material framework provide access to a diverse set of compositions. These material structures are formed from MX_6_ octahedra sharing corners, edges, or faces to form three-dimensional structures, two-dimensional systems, one-dimensional chains, or zero-dimensional clusters that are separated by organic cations [5,6,7,8]. Among these materials, the halogenostannate hybrid compounds containing protonated amine cations draw considerable attention, because of their interesting physical and chemical properties such as magnetic, electroluminescence, photoluminescence, and conductivity, which potentially lead to technological advances [9,10,11,12]. Apart from that, these compounds have gained attention due to their interesting antibacterial and antifungal properties against a wide range of bacterial and fungal species [13,14,15,16,17,18,19]. Several studies with fascinating results on antitumor activities against a diverse panel of human tumor cell lines have been reported [20,21,22]. In general, the structure of these compounds and the type of organic cations have a significant impact on their biochemical activity.

To date, many halogenostannate hybrid materials have been successfully assembled. However, there is a dearth of research on the interactions between organic and inorganic units. In comparison with other organic–inorganic coordination compounds, the rational design and assembly of organic halogenostannate, especially non-covalent connection, remains a difficult task for halogenostannate chemists. Several factors must be considered to achieve this goal, including the coordination geometry of the metal ions, the nature of the organic ligands, the solvent, pH, and reaction temperature. Ligand selection is the most crucial of these factors.

**Scheme 1:**
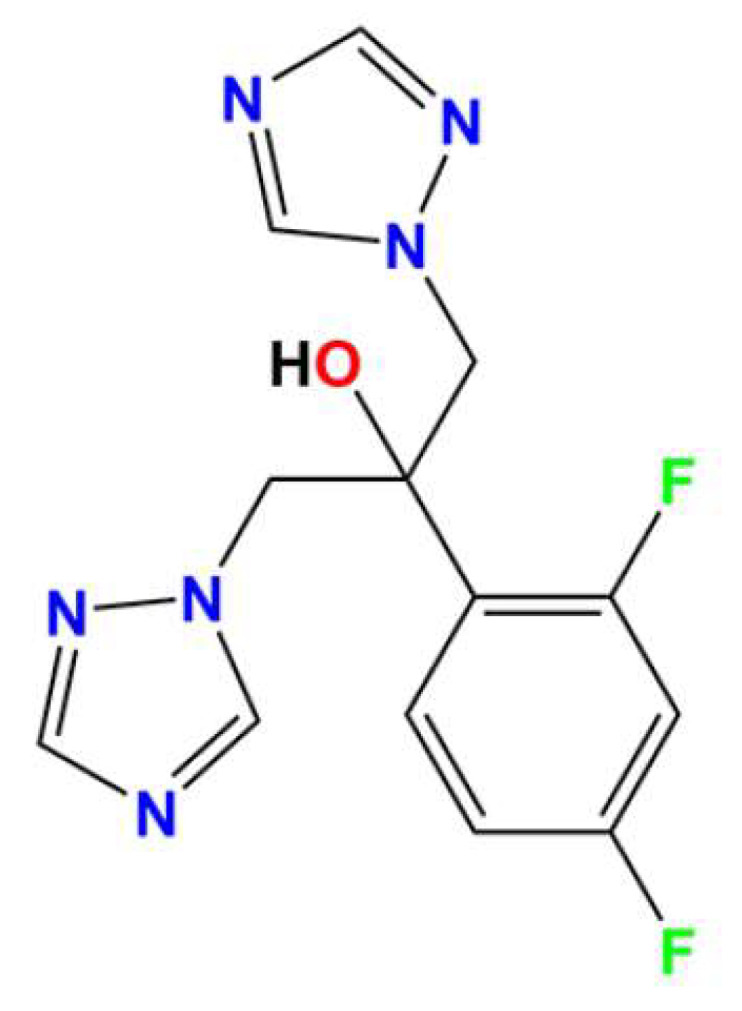
Molecular structure of fluconazole.

Fluconazole (1-(2,4-difluorophenyl)-1, 1-bis [(1H-1,2,4-triazol-1-yl) methyl] ethanol) (Scheme 1) is a 1,2,4-triazole derivative that is a commonly used as antifungal medicine but also a good flexible ligand to build metal–organic materials with optical properties and medical applications [23,24]. This ligand has several donor/acceptor groups (difluoro phenyl, triazole, and hydroxyl groups) that can form non-covalent interactions to stabilize the supramolecular architecture. A search in the Cambridge Structural Database (CSD version 5.42, November 2020) revealed 128 entries relating to metal complexes including fluconazole, compared to 112 entries relating to fluconazole molecules directly linked to the metal, with one structure reported by our group as the first organic–inorganic (linked by non-covalent interactions) salt base on bismuth-fluconazole [25].

In the context of this current study, we report the synthesis, detailed crystallographic analysis, vibrational study, and the optical and magnetic properties of a new hybrid organic–inorganic salt, (C_13_H_14_F_2_N_6_O).SnCl_6_.2H_2_O. In addition, this study is supported by an extensive computational investigation using the density functional theory (DFT) and time dependent density functional theory (TD-DFT). In the first step, we performed a theoretical benchmark that is more complete than any previously published data of this kind of systems, from the point of view of the number of functionals evaluated. Then, we retained the most suitable one to investigate the optical and electronic properties, as well as the frontier molecular orbitals (FMOs) and the energy gap between the highest occupied molecular orbital (HOMO) and the lowest unoccupied molecular orbital (LUMO). Then, we tried, to study the Cl-substitution effect to predict the gap energy and chemical descriptor.

## 2. Results and Discussion

### 2.1. Crystal Structure of (H2Fluconazole).SnCl6.2H_2_O

The single-crystal data and the refinement results are presented in Table 1. Atomic coordinates and equivalent isotropic displacement parameters are provided in Table S1 in the Supporting Information (SI). Selected interatomic distances and bond angles are presented in Table S2 in the Supporting Information (SI).

**Figure 1:**
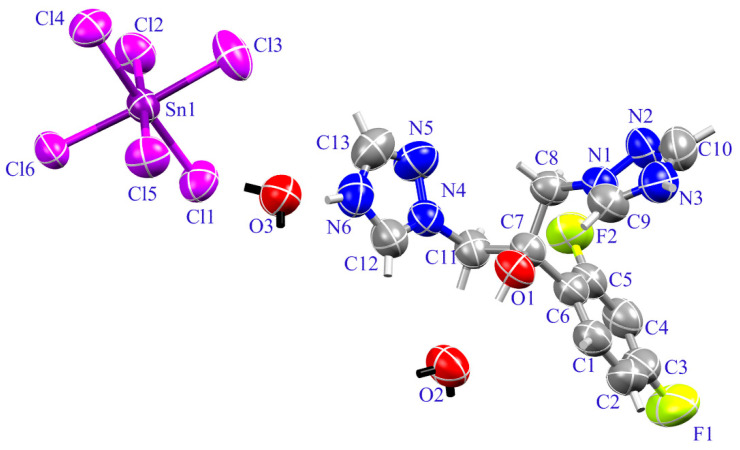
Asymmetric unit of (H2Fluconazole).SnCl6.2H_2_O with 50% probability ellipsoids.

**Figure 2:**
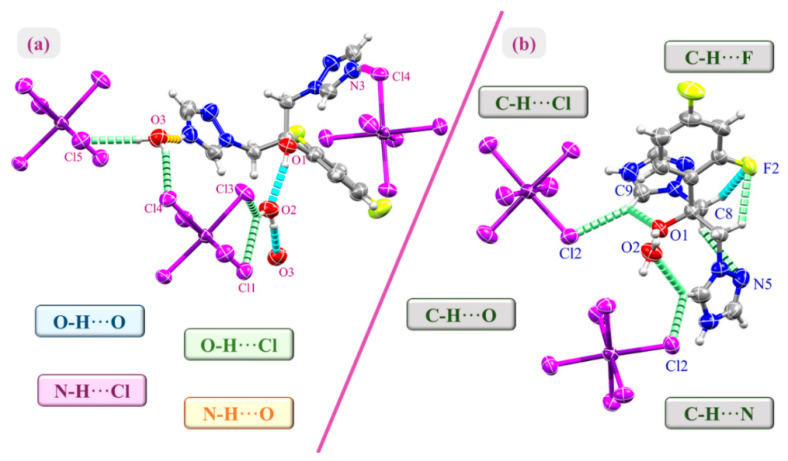
Hydrogen bond interactions in (H2Fluconazole).SnCl_6_.2H_2_O.

**Figure 3:**
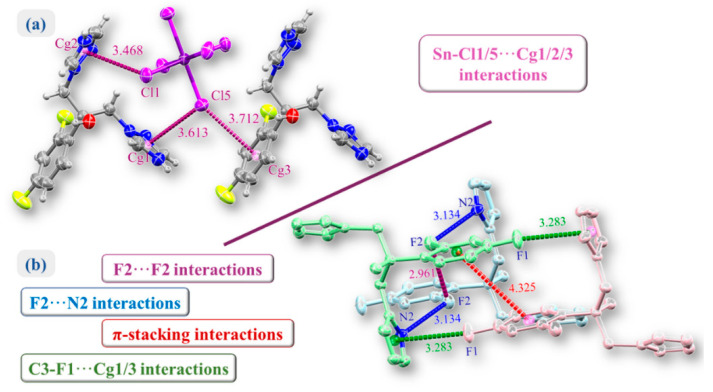
Details of the halogen bonding, X···π, π-stacking interactions in (H2Fluconazole).SnCl_6_.2H_2_O (with 50% and 20% probability ellipsoids in (**a**) and (**b**), respectively).

**Figure 4:**
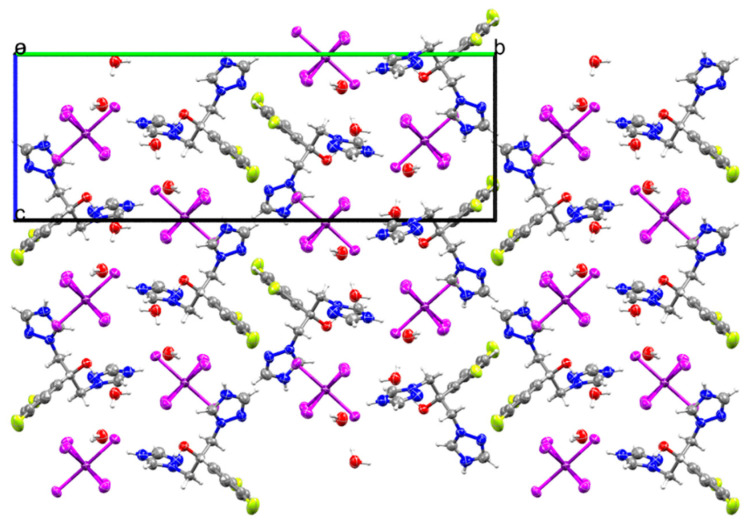
Overall packing of unit cell of (H2Fluconazole).SnCl_6_.2H_2_O viewed along the c-axis.

The (H2Fluconazole).SnCl_6_.2H_2_O salt crystallizes in the monoclinic system *P2_1_/n* space group with one anion [SnCl_6_]^2−^, one (H2Fluconazole)^2+^ cation and two water molecules in the asymmetric unit (Figure 1). Table S1 lists some selected bond lengths and angles of this structure. These bond lengths and angles are in good agreement with those observed in similar compounds based on fluconazole cation and hexachlorostannate (IV) [23,26,27,28,29]. The (H2Fluconazole)^2+^ cations interact with [SnCl_6_]^2−^ anions and the water molecules through strong intermolecular O-H···O (cyan dashed lines), N-H···O (orange dashed lines) and N-H···Cl (purple dashed lines) hydrogen bonds as shown in Figure 2a (Table 2). Additionally, the water molecule is bonded to the adjacent [SnCl_6_]^2−^ anions through O-H···Cl (Green dashed lines) hydrogen bonds (Table 2, Figure 2a). In addition to these strong hydrogen bonds, the cations are connected to the adjacent anions and water molecules via weak C-H···X (X = Cl and O) contacts (green dashed lines in Figure 2b). In this structure, H2Fluconazole randomly adopts anti-gauche (A-G) conformations [30]. The existence of intramolecular C-H···X (X = F, O and N) hydrogen bonds causes this conformation (Table 2, Figure 2b). Figure 3a shows how [SnCl_6_]^2−^ anions interact with H2Fluconazole cations via Cl1/5···Cg1/2/3 interactions. The d(Cl···Cg) distances are dCl1···Cg2 = 3.468 (2) Å, dCl5···Cg1 = 3.613 (2) Å and dCl5···Cg3 = 3.712 (2) Å, where Cg1 and Cg2 are the centroids of the triazole rings and Cg3 is the centroid of difluophenyl ring (purple dashed lines). Moreover, due to the inversion center, the H2Fluconazole cations are related to each other by halogen bonding, F···π, and π-stacking interactions. However, weak π-stacking interactions (Cg3···Cg3 = 4.325 (2) Å) were observed between the centroids of the difluorophenyl rings of the (H2Fluconazole) cations (red dashed lines in Figure 3b). In addition, the fluorine atom forms weak halogen bonds. From a geometric point of view, these F···F contacts can be classified as type I (d(F2···F2 = 2.961 (3) Å, Ang(C-F···F) = 100.80(16)°) purple dashed lines in Figure 3b) [31,32,33,34,35,36]. Additionally, the F atoms engage in a non-covalent interaction with the nitrogen atoms of the nearby. H2Fluconazole cations (d(F2···N2 = 3.134 (4) Å; blue dashed lines in Figure 3b). Furthermore, through C-F···π interactions, the fluorine atom interacts with adjacent triazole and difluorophenyl rings (dF1···Cg1 = 3.283 (3) Å, and dF1···Cg3 = 3.552 (3) Å, green dashed lines in Figure 3b). According to the geometry, the C-F bond is perpendicular or parallel to the aromatic ring, or, as in this instance, the C-F···π interaction take the form of T-shaped halogen bonding with Cg1 (C-F1···Cg1 = 171.31°) and π···π (lone-pair···π) with Cg3 (C-F1···Cg3 = 85.3°). Calculation on the electrostatic map was performed at the HSE theoretical level (Figure S1). This figure confirms the below Hirshfeld surface analysis and enrichment ratio calculation. indeed, we can see that the red region is the negative extreme which is mainly located in the inorganic part and the blue is the positive extreme and located in the organic part with slightly positive region located in the center of the difluorphenyl group interacting with the negative equatorial region of F. Finally, the interplay between hydrogen and halogen bonding as well as F···π and π-stacking interactions results in the formation of endless zig-zag chains in the [100] direction (Figure 4).

### 2.2. Hirshfeld Surface Analysis and Contact Enrichment Ratio

**Figure 5:**
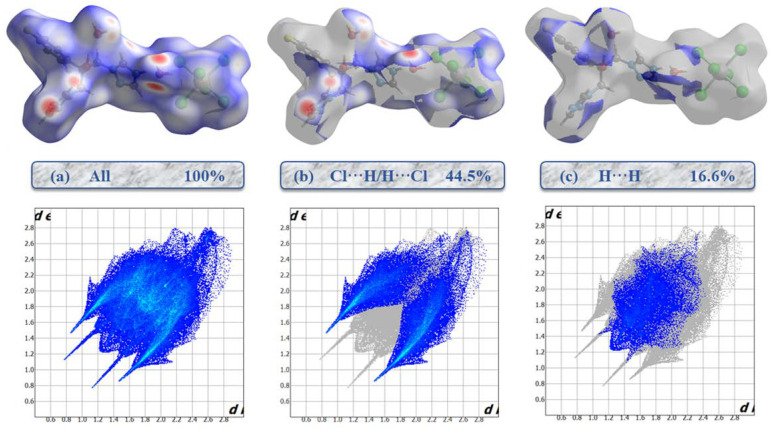
Two-dimensional fingerprint plots order with a d_norm_ view of (**a**) All (100%), (**b**) Cl⋯H/H⋯Cl (44.5%) and (**c**) H⋯H (16.6%) contacts in (H2Fluconazole).SnCl_6_.2H_2_O.

**Figure 6:**
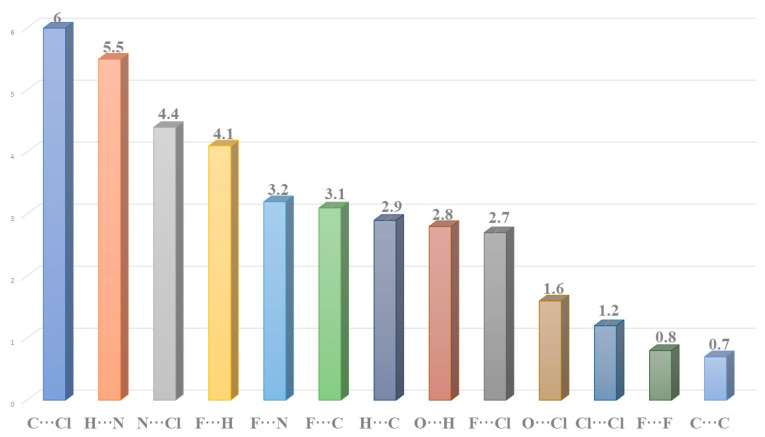
The relative contribution of intermolecular contacts in (H2Fluconazole).SnCl_6_.2H_2_O to the Hirshfeld surface.

The Hirshfeld surfaces mapped over d_norm_ as well as the 2D fingerprint plots of the abundant contacts in the structure are presented in Figure 5. It should be noted that regions of intense red color are located over the oxygen, carbon, and nitrogen atoms of organic cations and water molecules (Figure 5a). Hirshfeld surface (HFs) maps (Figure 5b) show that the most abundant contacts are the O/N/C-H···Cl hydrogen interactions, which account for 44.5% of the total HFs maps. The non-covalent H···H interactions occupy 16.6% of the total HFs maps indicating high contribution of triazole and difluorophenyl rings in the stabilization of the crystal packing (Figure 5c). The relative contributions of all contacts governing the structure are summarized in Figure 6.

The enrichment ratios (E), which are the ratios of actual contacts in the crystal to random contacts (theoretical proportion), were calculated using surface contact data from the Hirshfeld surface analysis, with E greater than one indicating a favored contact between pairs of elements and E less than one indicating a disfavored contact. The enrichment ratios of (H_2_Fluconazole).SnCl_6_.2H_2_O (Table 3) shows that the F⋯N halogen bonding and F⋯π interactions (E_FN_ = 3.33 and E_FC_ = 3.16) are the most favoured contact in the crystal packing followed by the H⋯Cl hydrogen bond contacts (E_HCl_ = 1.55), C⋯C π-stacking interactions (E_CC_ = 1.55), C⋯Cl contacts (E_CCl_ = 1.45), O⋯Cl (E_OCl_ = 1.18) and N⋯Cl contacts (E_NCl_ = 1.09), respectively. Theoretical and experimental equilibrium geometry of the (H2Fluconazole).SnCl6.2H2O listed in Table S4.

### 2.3. FT-IR and Raman Spectral Analysis

**Figure 7:**
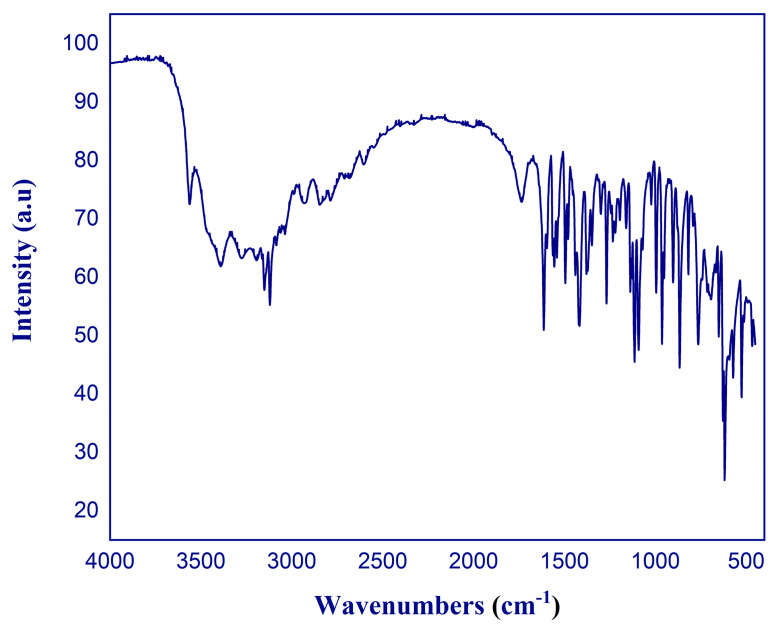
Experimental IR absorption spectrum of (H2Fluconazole).SnCl_6_.2H_2_O recorded at room temperature.

The solid-state IR and Raman spectra for (H2Fluconazole).SnCl_6_.2H_2_O (Figure 7 and Figure S2, respectively) are consistent with their crystal structure. Table 4 shows the band assignments in the IR and Raman spectra, which are based on the spectra from earlier studies of fluconazole cation and SnCl_6_ cluster [23,24,25,37,38,39] as well as quantum chemical computations.

#### Internal Modes of H2Fluconazole Cation

The stretching vibrations of the different functional groups interconnected by a series of hydrogen bonds in the crystal are represented in the high frequency region, which ranges from 3560 to 2600 cm^−^^1^. However, the presence of crystallization water in the structure is indicated by the strong signals centered at roughly 3560 and 3390 cm^−1^, which are assigned to the O-H stretching vibrations. The O-H stretching modes of the tertiary alcohol and N-H groups appeared as a weak band centered at 3274 and 3190 cm^−^^1^, respectively. In addition, the spectrum shows strong intensity bands in the 3150–3120 cm^−^^1^ spectral range which are attributed to C-H stretching modes of the triazole ring. These individual frequencies are slightly shifted because of the presence of intermolecular C-H/π interactions, as was discussed in the analysis of the crystal structure. The absorption bands of the ν_a_(CH2) and ν_s_(CH2) group in the complexes are observed in the frequency range 2928–2850 cm^−^^1^.

Asymmetric and symmetric bending vibrations of δ(N-H) and δ(C-H), as well as valence vibrations of ν(C-O), ν(C = C), ν(C-N), ν(C = N) and ν(C-F) generally appear coupled and can be observed in the 1607–1085 cm^−^^1^ range. Finally, N-H, O-H and C-H out-of-plane bending modes are attributed to the bands in the 1000–500 cm^−^^1^ range.

The scaled IR spectrum using HSEH1PBE functional associated to LANL2DZ pseudopotential and 6-311++G** basis in the considered region is in good agreement with the experimental data (Table 4).

### 2.4. UV–Visible Spectroscopic Study

**Figure 8:**
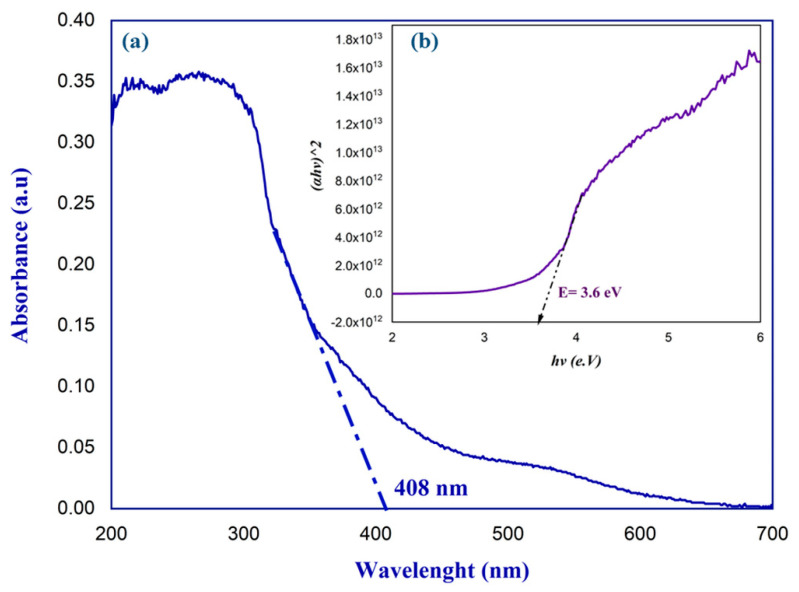
Solid-state UV-visible absorption spectra (**a**) and the Kubelka-Munk transformation of absorption data (**b**) of (H2Fluconazole).SnCl_6_.2H_2_O.

The optical absorption spectrum (300–700 nm) of (H2Fluconazole).SnCl_6_.2H_2_O (Figure 8a) reveals a broad absorption band across the ultraviolet region up to the absorption edge onset at 408 nm (3.03 eV). Two less pronounced bands can be seen in the range of 200–300 nm at 216 nm (5.74 eV) and 266 nm (4.66 eV). These two bands are produces by the charge transfer between the conjugated rings in H2Fluconazole [38,40].

The material’s diffuse reflectance was measured in order to estimate its optical band gap by changing the absorption spectrum into a Tauc plot Figure 8b, according to the Kubelka–Munk function: F(R) = (1 − R)^2^/2R, in which R is the experimentally observed reflectance. In a F(R)^2^ vs. photon energy plot, the energy band gap is determined as the point where the energy axis intersects with the extrapolated linear component of the absorption edge. The direct band gap value measured is 3.60 eV, which is typical with the compound’s semiconductor characteristics.

### 2.5. Magnetic Study

**Figure 9:**
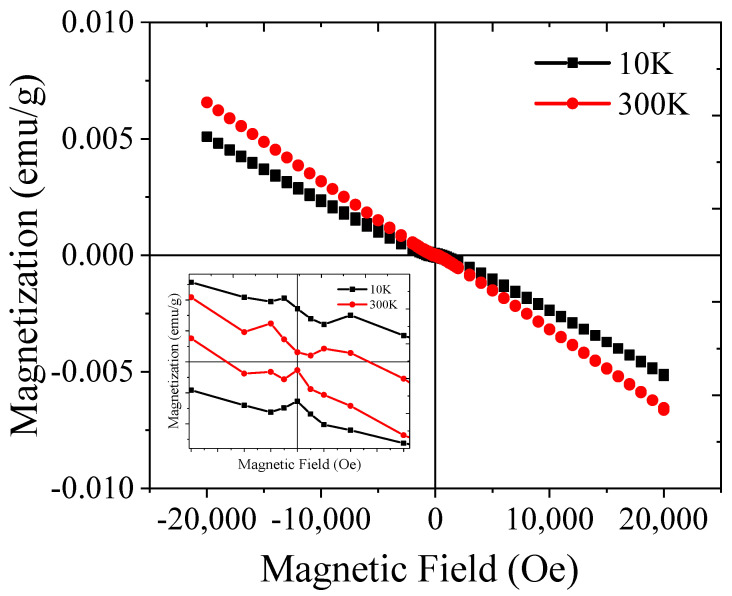
M-H curves at 10 and 300 K.

**Figure 10:**
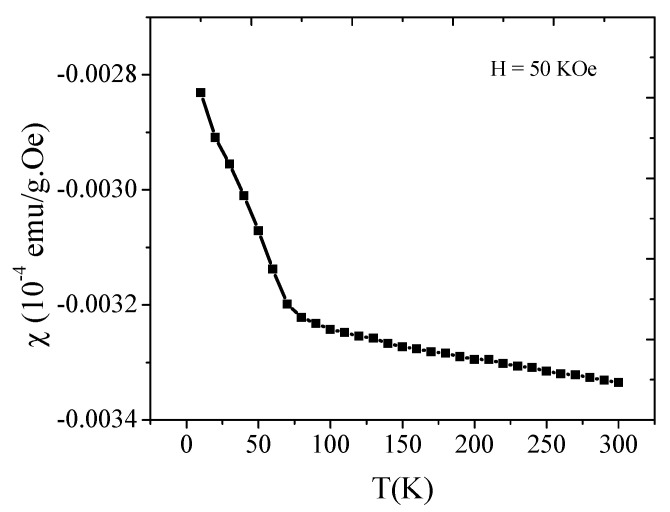
Magnetic susceptibility χ(T) measured at 50 KOe.

The magnetic hysteresis loops measured at 10 and 300 K are shown in Figure 9. The magnetization curves indicate the co-existence of two components: weak ferromagnetic and large diamagnetic behavior. The M-H curve at room temperature shows mainly a diamagnetic contribution with small magnetic susceptibility. The diamagnetic contributions (i.e., the linear part) at high magnetic field and the ferromagnetic behavior as shown in the inset of Figure 9. The temperature dependence of the magnetic susceptibility measured at H = 50 kOe is shown in Figure 10. The diamagnetic behavior is confirmed again by the negative magnetic susceptibility at high field. It can be concluded from the magnetic measurement that the synthesized 0D organic–inorganic hybrid material behaves as a diamagnetic material, with very low magnetic susceptibility. Previous reports showed a ferromagnetic behavior for two-dimensional organic–inorganic hybrids [41,42].

**Figure 11:**
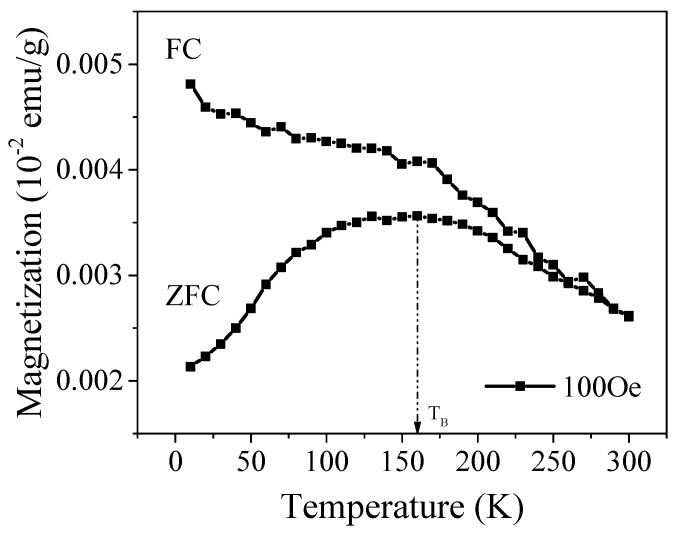
ZFC/FC curves.

To reveal the origin of the ferromagnetism in this sample, the magnetization as a function of temperature measured in the field-cooled (FC) and zero-field-cooled (ZFC) configurations at an applied magnetic field of 100 Oe was measured as shown in Figure 11.

The FC and ZFC curves show obvious bifurcation, which indicate the existing of magnetic nano clusters with blocking temperature of 160 K. Furthermore, the magnetization is not inversely proportional to T in the ZFC plot, indicating the non-existence of superparamagnetic nanoparticles. Therefore, the overall magnetization contributed to this system is originated from two sources. The first source of magnetization is the free carriers located in defect states where the density of magnetic contribution increases with the increase in localized carriers at low temperature. The second source of magnetization is the contribution of the magnetic nanoparticle exist in this sample as shown in the ZFC/FC measurements.

### 2.6. TD-DFT Study

Despite the difficult performance of benchmarks for this kind of systems, especially due to the requested resources, TD-DFT methods have become useful tools used by theorists and experiments to examine, understand, and predict the optical and chemical behavior of different types of organic and inorganic compounds [43,44,45,46] due to the remarkable ratio of accuracy and computational time.

As mentioned above, we have used nine density functionals to investigate the optical and chemical behavior of the studied compound.

In the first step, we focused on the evaluation of different functional performance by the comparison of calculated energy gap (Eg) with the experimental value (3.6 eV).

**Figure 12:**
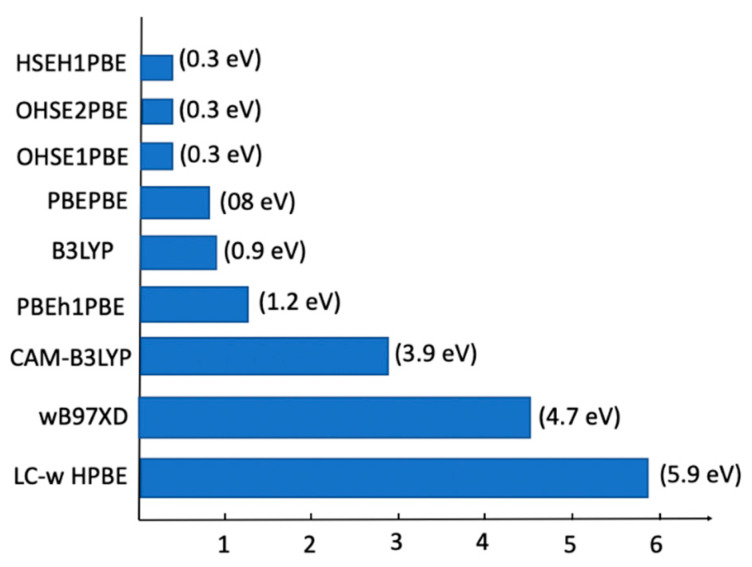
Absolute error between calculated energy gap (Eg) using different functionals and experimental value (3.6 eV).

As we see in Figure 12, the B3LYP, CAM-B3LYP, LC-wHPBE and wB97XD functionals fail to recover the correct Eg. This is mainly due to the weak bonds of water molecules.

For the five functionals employing PBE correlation, PBE1PBE underestimates the Eg by 0.8 eV while PBEh1PBE overestimates it by 1.2 eV. However, the OHSE1PBE, OHSE2PBE and HSE1PBE functionals effectively reproduce the energy gap with error at approximately 8% (3.9 vs. 3.6 eV).

Since the HSE1PBE functional is the recommended one for the study of this type of systems, it will be retained in the next step for the description of the electron excited state transitions, the examination of the different chemical reactivity descriptors and the prediction of the halogen-substitution effect on the chemical behavior of studied compound.

To investigate the electronic and optical properties, as well as the UV-visible spectra and the way that any molecule interacts with other species, is related to the inspection of the highest occupied molecular orbital (HOMO) and the lowest unoccupied molecular orbital (LUMO). The higher energy of LUMO as an electron acceptor represents the higher resistance to accept an electron. While the low energy of the HOMO indicates the lower ability to donate an electron. The UV–visible spectra were calculated using the HSE1PBE functional and listed in Table S3. Close inspection of the properties of the electronic transitions shows that the first transition is mainly the promotion of a single electron from the HOMO to the LUMO with a predicted oscillator strength of 0.0012. The second transition occurs in UV, with a relatively high oscillator strength calculated to be 0.0265. Finally, a transition in the UV region calculated to occurs at 219 nm. This latter is a combination from a promotion of one electron from HOMO−10 to LUMO+2 and a second transition from HOMO−8 to LUMO+2. It should be noted that theoretical calculations are in good agreement with those measured experimentally in UV the region, whereas we note a low accuracy in the near UV-visible region. This may be due to the multireference character of the transition.

To better understand and predict the chemical behavior of molecules, several chemical reactivity descriptors, which been have detailed in our previous works [25,47], are widely used.

These descriptors are related to frontier molecular orbitals (FMOs) and evaluated using HOMO and LUMO energy values according to the following equations [48,49]:

Energy gap (Eg), Eg = E(LUMO) − E(HOMO)(1)

First Ionization energy (I), I = −E_(HOMO)_ (2)

Affinity (A), A = −E_(LUMO)_ (3)

(4) $$\mathrm{Chemical}\;\mathrm{hardness}\;(\eta ),\;\eta =\frac{\left(E_{\mathrm{LUMO}}-E_{\mathrm{HOMO}}\right)}{2}$$

(5) $$\mathrm{Chemical}\;\mathrm{potential}\;(µ),\;µ=\frac{\left(E_{\mathrm{HOMO}}+E_{\mathrm{LUMO}}\right)}{2}$$

(6) $$\mathrm{Electrophilicity}\;(\omega ),\;\omega =\frac{{µ}^{2}}{2\eta }$$

(7) $$\mathrm{Electronegativity}\;(\chi ),\;\chi =\frac{\left(I\;+\;A\right)}{2}$$

(8) $$\mathrm{Softness}\;(S),\;S=\frac{1}{2\eta }$$

The energy gap (Eg) between the HOMO and the LUMO is a critical parameter to demonstrate the bioactivity and the intermolecular charge transfer [50]. A molecule with a small Eg is more polarizable and is generally associated with high chemical reactivity and low kinetic stability due to ease of charge transfer. It is also a soft molecule, which is confirmed by the low softness (S) value.

The low Eg of (H2Fluconazole).SnCl_6_.2H_2_O (Eg = 3.6 eV) is a proof of the intramolecular charge transfer (ICT), which supports the antioxidant ability of the studied compound.

We will now examine the Cl-substitution effect on the electronic properties, that is, the exchange of Cl by F, Br or I. The main electronic data for the different. (H2Fluconazole).SnCl_6_.2H_2_O compounds are reported in Table 5. At first glance, it can be seen that Eg decreases with the atomic number of halogens.

As a large Eg is preferred for higher stability, we can conclude that the compound resulting from the Cl substitution by F is more stable and thus less reactive. The Cl substitution by I leads to a less stable compound and eventually more important intramolecular charge transfer within the molecule and so a significant antioxidant ability.

This trend was is consistent with those of other chemical reactivity descriptors.

**Figure 13:**
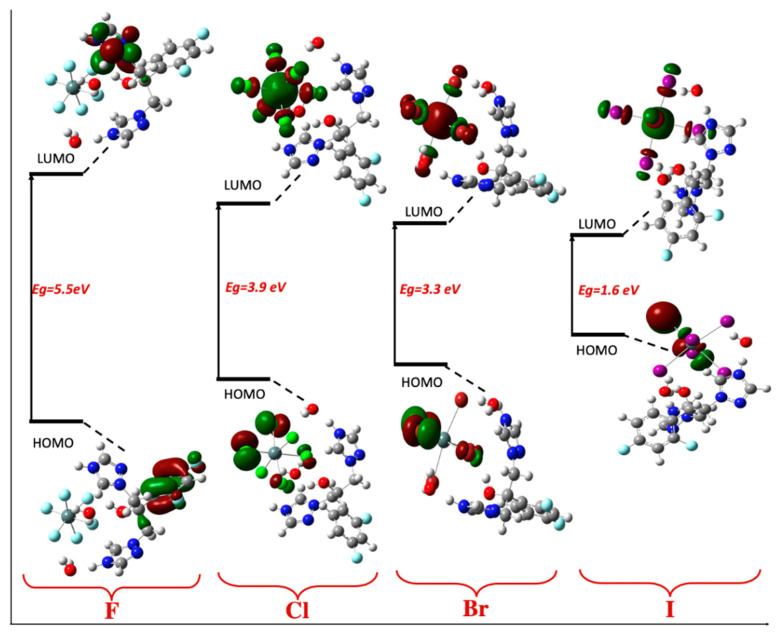
Frontier molecular orbitals and energy gap (Eg in eV) of (H2Fluconazole).SnX_6_.2H_2_O; (X = F, Cl, Br and I).

The contour surfaces of the FMOs are depicted in Figure 13. Differently to Br and I, in the case of the compound resulting from the Cl substitution by F, the HOMO and the LUMO are localized on the organic cation. More precisely, the electron concentration of HOMO is on the 2,4-difluorophenyl ring of the H2Fluconazolium molecule, while the LUMO is localized on one of the triazolium rings without any electron concentration on inorganic anion.

In the case of (H2Fluconazole).SnX_6_.2H_2_O; (X = Cl, Br, I) compounds, the HOMO and the LUMO are localized only in the inorganic anion. Hence, Eg and other chemical reactivity descriptors are primarily linked to the inorganic anion due its importance in the activity of the molecule.

## 3. Materials and Methods

### 3.1. Salt Preparation

All the starting reagents were acquired commercially and were used without further purification. All the synthesis processes were carried out in the open air. A mixture of tin chloride (1 mmol, 0.074 g) and fluconazole (2 mmol, 0.1 g) was dissolved in concentrated hydrochloric acid (10 mL) and stirred for 2 h at 60 °C. The saturated colorless solution was slowly cooled and evaporated at ambient temperature. Colorless crystals suitable for X-ray diffraction analysis were obtained after 1 day.

### 3.2. X-ray Structure Determination

Single-crystal X-ray diffraction experiments were carried out on a STOE IPDS 2 diffractometer equipped with a graphite monochromator. Data collection and unit cell refinement for the datasets were performed using the X-AREA, data reduction and integration were performed by X-RED32 [51]. The crystal structures were solved by using Olex2 [52] software and the model obtained was refined by full-matrix least-squares on F2 (SHELXL2018/3) [53].

Anisotropic displacement parameters were used to refine all non-hydrogen atoms. A riding model was used to place hydrogen atoms at calculated positions. The new salt structure’s crystallographic information was deposited in the CSD [54] with the code CCDC 2012995.

### 3.3. Physicochemical Characterization

The solid-state infrared spectrum was recorded using a Bruker Tensor 27 FT-IR spectrometer with scanning range set from 400 to 4000 cm^−1^. The room temperature Raman spectrum was recorded in the region of 500–100 cm^−1^ with a RENISHAW RAMAN in Via Microscope with an excitation wavelength of 532 nm. The room-temperature UV–visible absorption spectrum of the polycrystalline powder of (H2Fluconazole).SnCl_6_.H_2_O was measured using a PerkinElmer Lambda 750 spectrophotometer in the wavelength range 300–700 nm, and the BaSO4 plate was used as reference. Magnetic characterizations were performed in a Quantum Design MPMS-5S SQUID magnetometer. Zero-field-cooled and field-cooled (ZFC-FC) curves were recorded at a magnetic field of 100 Oe.

### 3.4. Hirshfeld Surface Analysis (HS)

Hirshfeld surface analysis is a useful approach for evaluating weaker intermolecular interactions such as hydrogen and halogen bonds and other weak contacts. The Hirshfeld surface [55] and the decomposed two-dimensional (2D) fingerprint plots [55] were computed with CrystalExplorer17 [56]. Furthermore, the enrichment ratios (E) [57] of the intermolecular contacts were calculated to estimate the likelihood of two chemical species interacting.

### 3.5. Theoretical Calculations

Computations have been carried out with the Gaussian 16 program [58]. The asymmetric unit of (H2Fluconazole).SnCl_6_.2H_2_O crystal (Figure 1) was used to start the geometry optimization and frequency calculations. To perform our benchmark, we have employed several hybrid functionals. We started by using the famous Becke Three-Parameter Hybrid functional (B3LYP) [59,60], then we tested two long-range corrected functionals-CAM-B3LYP [61] and LC-wHPBE [62,63]. We also employed the wB97XD [64] functional including the dispersion correction (Grimme’s D2 dispersion model). In addition, we tested five functionals employing PBE correlation-PBE1PBE [65] and PBEH1PBE [66]; the HSE functionals OHSE1PBE and OHSE2PBE; and the recommended version of the full Heyd-Scuseria-Erzerhof functional HSE1PBE given by Scuseria and coworkers [67,68,69,70,71], which have been proved as suitable functionals for solid-state calculations. In these calculations, the LANL2DZ pseudopotential [72] was used to describe the Sn and I atoms and the 6-311++G** basis set was used to describe F, O, N, C, H, Cl and Br atoms.

To treat all the elements on an equal footing, additional calculations with the 6-31+G* basis set for the light atoms and LANL2DZ for the Sn atom were performed. The quality of the basis set does not affect the UV-visible spectrum and the different chemical reactivity descriptors studied. All geometries were fully optimized in the C1 symmetry group. All structures present a real minimum in their potential energy surface, and this was confirmed by the lack of imaginary frequencies.

TD-DFT calculations were performed at the optimized geometries using the same levels of theory. These extensive calculations were performed to examine the optical behavior, the chemical reactivity descriptors and the reliability of various functionals.

## 4. Conclusions

In this work, a new salt of the antifungal drug fluconazole, (H2Fluconazole).SnCl_6_.2H_2_O, was synthesized and characterized by single-crystal XRD, Hirshfeld surface analysis, Raman, FT-IR and UV-visible spectroscopies. This new compound crystallizes with a monoclinic crystal structure in the *P2_1_/n* space group and represents an 0D structure built of alternating organic and inorganic zig-zag layers along the crystallographic c-axis. The nature of intermolecular interactions in the title compound was also confirmed and quantified using HS analysis and enrichment ratio calculations. IR and Raman techniques confirm all vibrational modes of molecular groups. The optical properties of (H2Fluconazole).SnCl_6_.2H_2_O at the absorption edge revealed less pronounced bands at 216 and 266 nm, respectively. The direct band gap energy measured was 3.6 eV. The magnetic studies show that at low magnetic fields, weak ferromagnetic contributions coexist with large diamagnetic contributions, implying that the synthesized material behaves primarily as a diamagnetic material, with very low magnetic susceptibility. Despite the extensive DFT calculations in conjunction with large basis sets, only the HSE functionals, i.e., OHSE1PBE, OHSE2PBE and HSE1PBE, reproduce the Eg effectively. From a comparative point of view, HSE1PBE agrees better with the measured experimental values, where the largest error is approximately 16% in the H-O bond. According to our study, we recommend the HSE1PBE functional to study similar systems. HSE1PBE provides accurate data about the antioxidant ability of the synthetized crystal and predicts the Cl-substitution effect on the chemical behavior. A significant intermolecular charge transfer (ICT) was found in the case of Cl, Br and I. Finally, FMOs show that the ICT process is mainly linked to the inorganic anion part.

## Figures and Tables

**Table 1:** Crystallographic data and structure refinement parameters of (H2Fluconazole).SnCl_6_.2H_2_O.

Empirical Formula	(C_13_H1_4_F_2_N_6_O).SnCl_6_.2H_2_O
Formula weight (g/mol)	675.72
Crystal system, space group	monoclinic, *P2_1_/n*
a (Å)	8.0328 (5)
b (Å)	29.4011 (13)
c (Å)	10.3323 (7)
β (°)	100.901 (5)
V (Å^3^)	2396.2 (2)
Z	4
µ (mm^−1^)	1.78
Dx (Mg m^−3^)	1.873
F(000)	1328
Crystal size (mm)	0.72 × 0.64 × 0.58
Crystal habit	Colorless, Prism
θmin/θmax (deg)	1.4/26.8
Measured reflections	13,858
Independent reflections	5043
Observed refl. with I > 2σ(I)	4495
Rint	0.046
Data/restraints/parameters	5043/0/289
R[F^2^ > 2σ(F^2^)]	0.037
wR (F^2^)	0.088
GooF = S	1.12
Δρmax/Δρmin (e.Å^−3^)	0.53/−0.95

**Table 2:** Intermolecular interactions in (H2Fluconazole).SnCl_6_.2H_2_O.

D-H···A	D-H (Å)	H···A (Å)	D···A (Å)	D-H···A(°)
O1-H1···O2	0.82	1.94	2.750 (3)	169
O2-H2A···O3 ^ii^	0.85	2.01	2.820(4)	160
O2-H2A···Cl1 ^ii^	0.85	2.81	3.522(3)	142
O2-H2B···Cl3 ^i^	0.85	2.77	3.395(3)	132
O3-H3A···Cl5	0.85	2.37	3.214(3)	177
C12-H12···Cl2 ^i^	0.93	2.72	3.359 (4)	127
C12-H12···O2	0.93	2.52	3.284 (4)	140
C11-H11A···F2	0.97	2.29	2.921 (4)	122
C11-H11B···Cl3 ^ii^	0.97	2.93	3.546 (3)	122
C8-H8A···F2	0.97	2.40	3.038 (4)	123
C8-H8B···Cl6 ^iii^	0.97	2.86	3.471 (3)	122
C8-H8B···Cl1 ^iii^	0.97	2.90	3.704 (3)	141
C8-H8B···N5	0.97	2.57	3.221 (5)	124
C9-H9···Cl2 ^iv^	0.93	2.70	3.566 (3)	155
C9-H9···O1	0.93	2.34	2.813 (4)	111
C10-H10···Cl6 ^v^	0.93	2.93	3.855 (4)	175
N6-H6···O3	0.85 (5)	1.91 (5)	2.736 (4)	162 (6)
N3-H3···Cl4 ^iv^	0.85 (5)	2.44 (5)	3.240 (3)	157 (4)

Symmetry codes: (i) x − 1, y, z; (ii) x − 1/2, −y + 1/2, z + 1/2; (iii) x − 1/2, −y + 1/2, z − 1/2; (iv) x − 3/2, −y + 1/2, z − 1/2; (v) −x + 3/2, y + 1/2, −z + 1/2.

**Table 3:** Analysis of contacts on the Hirshfeld surface. The % of contact types (SX) between chemical species is given followed by random contacts (%) and their enrichment ratio (E). The major contacts as well as the major enriched ones are highlighted in bold characters.

Atoms	Cl	H	C	N	O	F
**(%) Contacts from fingerprint plots**						
**Cl**	1.2	-	-	-	-	-
**H**	44.5	16.6	-	-	-	-
**C**	6	2.9	0.7	-	-	-
**N**	4.4	5.5	0	0	-	-
**O**	1.6	2.8	0	0	0	-
**F**	2.7	4.1	3.1	3.2	0	0.8
**Surface (%)**	**30.8**	**46.5**	**6.7**	**6.54**	**2.2**	**7.35**
**Random contacts % (R_XX_ and R_XY_)**						
**Cl**	9.84	-	-	-	-	-
**H**	28.64	21.6	-	-	-	--
**C**	4.13	6.32	0.45	-	-	-
**N**	4.03	6.09	0.88	0.043	-	-
**O**	1.35	2.05	0.29	0.28	0.048	-
**F**	4.52	6.84	0.98	0.96	0.32	0.54
**Enrichment ratio E (E_XX_ and E_XY_)**						
**Cl**	0.13	-	-	-	-	-
**H**	**1.55**	0.77	-	-	-	-
**C**	**1.45**	0.46	**1.55**	-	-	-
**N**	**1.09**	0.9	0	0	-	-
**O**	**1.18**	**1.36**	0	0	0	-
**F**	0.59	0.6	**3.16**	**3.33**	0	

**Table 4:** Attribution of the experimental and calculated IR frequencies (cm^−1^) of (H2Fluconazole).SnCl_6_.2H_2_O.

Experimental	HSEH1PBE	Attribution
495-547	450-572	skeletal deformation
613	611	C-H out of plane
677	695	O-H out of plane
749	755	N-H out of plane of triazole group
837	818	C-H bending of triazole
904	897	C-H bending of benzene
948	965	C-C bending
1004	1001	C-N bending
1071	1085	C-O stretch mode
1103	1115	C-H and N-H bending of triazole
1257	1278	C-F bending mode
1408	1415	C-N stretch of triazole ring
1479	1490	C = C stretch 2,4-difluorobenzyl group
1530	1554	C = N stretch of triazole ring
1620	1607	C = N stretch of triazole ring
1631	1740	Benzene ring deformation
2411	2850	N-H stretch of triazole ring
2989	2928	C-H stretch of propane backbone
3156	3120	C-H stretch of propane backbone
3339	3150	C-H stretch (antisymmetric) of propane backbone
3412	3190	N-H stretch of triazole
3497	3274	O-H stretch mode
3603	3390	νs(OH) of crystallization water
3607	3560	νas(OH) of crystallization water

**Table 5:** Global reactivity descriptor values (in eV) of (H2Fluconazole).SnX_6_.2H_2_O; (X = F, Cl, Br, and I).

	F (Z = 9)	Cl (Z = 17)	Br (Z = 35)	I (Z = 53)
**ELUMO**	−1.9	−3.2	−3.4	−4.3
**EHOMO**	−7.4	−7.2	−6.5	−6.0
**Eg**	5.5	3.9	3.1	1.6
**Ionis**	7.4	7.2	6.5	6.0
**Elec. Aff**	1.9	3.2	3.4	4.3
**Hardness**	2.7	2.0	1.6	0.8
**Softness**	0.2	0.3	0.3	0.6
**Chem. Pot**	−4.7	−5.2	−5.0	−5.2
**Electrophilicity**	4.0	6.9	7.8	16.5
**Electronegativity**	4.7	5.2	5.0	5.2

## Data Availability

The raw/processed data generated in this work are available upon request from the corresponding author.

## Supplementary Materials

The following supporting information can be downloaded at: https://www.mdpi.com/article/10.3390/ijms23158765/s1.
