# Supplementary material for: New Organic–Inorganic Salt Based on Fluconazole Drug: TD-DFT Benchmark and Computational Insights into Halogen Substitution
Source: Int J Mol Sci. 2022 Aug 6;23(15):8765. doi: 10.3390/ijms23158765 (PMC9369134; doi:10.3390/ijms23158765)
Supplement: Supplementary file 1 [file ijms-23-08765-s001.zip › ijms-1824301-supplementary.pdf]

Supplementary materials of New organic-inorganic salt based on fluconazole drug: TD-DFT Benchmark and computational insights into halogen substitution

Ferjani Hela, Rim bechaieb, M. Alshammari, O. M. Lemine, and Necmi Dege

Corresponding Author: Ferjani Hela

E-mail address: hhferjani@imamu.edu.sa

Table S1

Fractional atomic coordinates and isotropic or equivalent isotropic displacement parameters (Å^2)

	x	*y*	*z*	U_{iso}*/U_{eq}
**Sn1**	1.18781 (3)	0.14677 (2)	0.47919 (2)	0.03890 (8)
**Cl6**	1.25999 (11)	0.08587 (3)	0.63572 (9)	0.05174 (19)
**Cl1**	1.01125 (11)	0.18582 (3)	0.60870 (9)	0.0543 (2)
**Cl4**	1.35721 (11)	0.10812 (3)	0.33621 (8)	0.0534 (2)
**Cl2**	1.43827 (10)	0.18700 (3)	0.58938 (9)	0.05293 (19)
**Cl5**	0.94373 (11)	0.10427 (3)	0.37042 (9)	0.0559 (2)
**Cl3**	1.13177 (14)	0.20755 (3)	0.31815 (9)	0.0637 (2)
**F2**	0.5046 (2)	0.46091 (7)	0.5909 (2)	0.0590 (5)
**O1**	0.2655 (3)	0.35109 (7)	0.3613 (2)	0.0472 (5)
**N1**	0.3370 (3)	0.42854 (8)	0.2208 (3)	0.0403 (5)
**F1**	−0.0055 (4)	0.49542 (9)	0.7217 (3)	0.0903 (9)
**N4**	0.5973 (3)	0.32775 (9)	0.4857 (3)	0.0452 (6)
**O2**	0.1788 (3)	0.29241 (8)	0.5450 (3)	0.0635 (7)
**O3**	0.6540 (3)	0.18062 (8)	0.3026 (3)	0.0667 (7)
**N2**	0.3685 (4)	0.47119 (9)	0.1776 (3)	0.0535 (7)
**N6**	0.6745 (4)	0.26532 (11)	0.4142 (3)	0.0560 (7)
**N3**	0.1518 (4)	0.44026 (11)	0.0493 (3)	0.0546 (7)
**C6**	0.2626 (4)	0.41760 (10)	0.5007 (3)	0.0407 (6)
**N5**	0.7466 (4)	0.33679 (12)	0.4459 (3)	0.0595 (7)
**C5**	0.3360 (4)	0.45267 (10)	0.5814 (3)	0.0448 (7)
**C12**	0.5553 (5)	0.28516 (12)	0.4662 (4)	0.0559 (8)
**C7**	0.3672 (4)	0.38537 (10)	0.4320 (3)	0.0402 (6)
**C11**	0.5036 (4)	0.36322 (10)	0.5391 (3)	0.0450 (7)
**C8**	0.4543 (4)	0.40911 (11)	0.3319 (3)	0.0436 (7)
**C4**	0.2512 (5)	0.47946 (11)	0.6561 (4)	0.0533 (8)
**C1**	0.0900 (4)	0.41137 (11)	0.4926 (4)	0.0484 (7)
**C3**	0.0815 (5)	0.47052 (12)	0.6457 (4)	0.0594 (9)
**C9**	0.2087 (4)	0.41008 (12)	0.1430 (4)	0.0514 (7)
**H9**	0.164965	0.381162	0.151257	0.062*

**C13**	0.7876 (4)	0.29782 (14)	0.4028 (4)	0.0617 (9)
**H13**	0.884670	0.292939	0.367820	0.074*
**C10**	0.2530 (5)	0.47684 (13)	0.0732 (4)	0.0592 (9)
**H10**	0.241459	0.502925	0.021367	0.071*
**C2**	−0.0018 (5)	0.43763 (12)	0.5654 (4)	0.0588 (9)

Atomic displacement parameters (Å^2)

	U11	***U22***	***U33***	***U12***	***U13***	***U23***
**Sn1**	0.04669 (13)	0.03443 (12)	0.03599 (12)	−0.00020 (8)	0.00889 (8)	0.00013 (7)
**Cl6**	0.0634 (5)	0.0432 (4)	0.0522 (5)	0.0073 (3)	0.0200 (4)	0.0117 (3)
**Cl1**	0.0611 (5)	0.0537 (4)	0.0501 (5)	0.0112 (4)	0.0154 (4)	−0.0059 (3)
**Cl4**	0.0574 (4)	0.0621 (5)	0.0435 (4)	0.0033 (4)	0.0166 (4)	−0.0077 (3)
**Cl2**	0.0552 (4)	0.0473 (4)	0.0537 (5)	−0.0094 (3)	0.0035 (4)	−0.0009 (3)
**Cl5**	0.0534 (4)	0.0558 (5)	0.0573 (5)	−0.0103 (4)	0.0077 (4)	−0.0102 (4)
**Cl3**	0.1003 (7)	0.0460 (4)	0.0413 (4)	0.0004 (4)	0.0041 (4)	0.0080 (3)
**F2**	0.0517 (10)	0.0549 (11)	0.0704 (14)	−0.0165 (9)	0.0111 (9)	−0.0154 (10)
**O1**	0.0577 (13)	0.0413 (12)	0.0417 (12)	−0.0112 (9)	0.0073 (10)	−0.0032 (9)
**N1**	0.0432 (12)	0.0411 (13)	0.0376 (13)	−0.0027 (10)	0.0099 (10)	0.0000 (10)
**F1**	0.1039 (18)	0.0678 (15)	0.118 (2)	0.0022 (13)	0.0691 (18)	−0.0207 (15)
**N4**	0.0491 (14)	0.0453 (14)	0.0416 (14)	0.0022 (11)	0.0097 (11)	0.0041 (11)
**O2**	0.0801 (17)	0.0470 (13)	0.0672 (17)	−0.0070 (12)	0.0238 (14)	0.0006 (12)
**O3**	0.0649 (15)	0.0568 (15)	0.083 (2)	0.0041 (12)	0.0257 (14)	0.0036 (13)
**N2**	0.0603 (16)	0.0411 (14)	0.0581 (18)	−0.0023 (12)	0.0089 (14)	0.0068 (12)
**N6**	0.0636 (18)	0.0497 (16)	0.0545 (18)	0.0105 (14)	0.0110 (14)	0.0005 (13)
**N3**	0.0513 (16)	0.0654 (19)	0.0448 (16)	0.0036 (14)	0.0031 (13)	0.0060 (14)
**C6**	0.0474 (15)	0.0373 (14)	0.0383 (15)	−0.0037 (12)	0.0102 (12)	0.0011 (12)
**N5**	0.0432 (14)	0.0671 (18)	0.068 (2)	−0.0047 (14)	0.0099 (14)	−0.0050 (16)
**C5**	0.0501 (16)	0.0391 (15)	0.0455 (17)	−0.0058 (12)	0.0101 (13)	0.0042 (12)
**C12**	0.064 (2)	0.0450 (18)	0.062 (2)	0.0035 (15)	0.0200 (17)	0.0076 (15)
**C7**	0.0453 (15)	0.0376 (14)	0.0374 (15)	−0.0050 (11)	0.0074 (12)	−0.0018 (11)
**C11**	0.0559 (17)	0.0419 (15)	0.0371 (16)	0.0025 (13)	0.0086 (14)	−0.0018 (12)
**C8**	0.0424 (15)	0.0494 (17)	0.0384 (16)	−0.0042 (12)	0.0066 (12)	0.0023 (12)
**C4**	0.072 (2)	0.0367 (16)	0.054 (2)	−0.0064 (15)	0.0190 (17)	−0.0054 (14)
**C1**	0.0500 (17)	0.0439 (16)	0.0523 (19)	−0.0067 (13)	0.0120 (14)	0.0014 (14)
**C3**	0.074 (2)	0.0447 (18)	0.069 (2)	0.0047 (16)	0.037 (2)	0.0013 (16)
**C9**	0.0547 (18)	0.0519 (18)	0.0465 (18)	−0.0080 (14)	0.0067 (15)	0.0003 (14)
**C13**	0.0476 (18)	0.074 (2)	0.063 (2)	0.0078 (17)	0.0090 (17)	−0.0067 (19)
**C10**	0.064 (2)	0.0536 (19)	0.059 (2)	0.0090 (16)	0.0084 (18)	0.0109 (16)
**C2**	0.0530 (18)	0.055 (2)	0.074 (2)	−0.0018 (16)	0.0246 (18)	0.0069 (18)

Table S2

Selected Bond Lengths (Å) and Bond Angles (°) for (H2Fluconazole)$SnCl_6.2H_2O$.

**Selected Bond lengths**			
**Sn1-Cl6**	**2.4090 (8)**	**N3-C9**	**1.329 (5)**
**Sn1-Cl5**	2.4156 (8)	**N3-C10**	1.343 (5)
**Sn1-Cl1**	2.4161 (8)	**C6-C1**	1.386 (4)
**Sn1-Cl2**	2.4242 (8)	**C6-C5**	1.386 (4)
**Sn1-Cl3**	2.4251 (9)	**C6-C7**	1.527 (4)
**Sn1-Cl4**	2.4669 (8)	**N5-C13**	1.295 (5)
**F2-C5**	1.360 (4)	**C5-C4**	1.371 (5)
**O1-C7**	1.411 (4)	**C7-C8**	1.523 (4)
**O1-H1**	0.8200	**C7-C11**	1.546 (4)
**N1-C9**	1.300 (4)	**N2-C10**	1.294 (5)
**N1-N2**	1.371 (4)	**N6-C12**	1.320 (5)
**N1-C8**	1.457 (4)	**N6-C13**	1.339 (5)
**F1-C3**	1.360 (4)	**C4-C3**	1.372 (5)
**N4-C12**	1.302 (4)	**C1-C2**	1.384 (5)
**N4-N5**	1.366 (4)	**C3-C2**	1.364 (6)
**N4-C11**	1.455 (4)		

**Selected Bond Angles**			
**Cl6-Sn1-Cl5**	**89.94 (3)**	**O1-C7-C8**	**105.7 (2)**
**Cl6-Sn1-Cl1**	94.21 (3)	**O1-C7-C6**	111.6 (2)
**Cl5-Sn1-Cl1**	89.64 (3)	**C8-C7-C6**	113.2 (2)
**Cl6-Sn1-Cl2**	88.53 (3)	**O1-C7-C11**	109.4 (2)
**Cl5-Sn1-Cl2**	178.03 (3)	**C8-C7-C11**	109.0 (2)
**Cl1-Sn1-Cl2**	91.72 (3)	**C6-C7-C11**	107.8 (2)
**Cl6-Sn1-Cl3**	176.56 (3)	**N4-C11-C7**	112.2 (3)
**Cl5-Sn1-Cl3**	91.89 (3)	**N1-C8-C7**	113.8 (2)
**Cl1-Sn1-Cl3**	88.72 (3)	**C5-C4-C3**	116.0 (3)
**Cl2-Sn1-Cl3**	89.58 (3)	**C2-C1-C6**	121.6 (3)
**Cl6-Sn1-Cl4**	88.26 (3)	**F1-C3-C2**	119.1 (3)
**Cl5-Sn1-Cl4**	88.52 (3)	**F1-C3-C4**	117.6 (4)
**Cl1-Sn1-Cl4**	176.92 (3)	**C2-C3-C4**	123.3 (3)
**Cl2-Sn1-Cl4**	90.18 (3)	**N1-C9-N3**	107.2 (3)
**Cl3-Sn1-Cl4**	88.87 (3)	**C1-C6-C7**	121.5 (3)
**C9-N1-N2**	110.7 (3)	**C5-C6-C7**	122.2 (3)
**C9-N1-C8**	130.0 (3)	**C13-N5-N4**	103.1 (3)
**N2-N1-C8**	118.9 (2)	**F2-C5-C4**	116.8 (3)
**C12-N4-N5**	110.9 (3)	**F2-C5-C6**	118.7 (3)
**C12-N4-C11**	127.6 (3)	**C4-C5-C6**	124.5 (3)
**N5-N4-C11**	121.5 (3)	**N4-C12-N6**	107.5 (3)
**C10-N2-N1**	104.0 (3)	**N5-C13-N6**	112.3 (3)
**C12-N6-C13**	106.1 (3)	**N2-C10-N3**	111.4 (3)
**C9-N3-C10**	106.7 (3)	**C3-C2-C1**	118.4 (3)
**C1-C6-C5**	116.2 (3)		

Table S3: Calculated UV-Vis spectrum, oscillator strength and the major contribution calculated using HSE1PBE functional.

λ_{cal} (nm)	Excitation energy (eV)	Oscillator strength	Major contributions
347.71	3.5657	0.0012	H -->L (0.705150
238.23	5.2043	0.0265	H-3 -->L+2 (0.60696)
219.39	5.6512	0.0126	H-10 -->L+2 (0.30488)<br>H-8 -->L+2 (0.45574)

Table S4: Theoretical and experimental equilibrium geometry of the (H2Fluconazole).$SnCl_6.2H_2O$

	HSE1PBE	Exp	Error %
Sn-Cl4	2.5692	2.4669	3.98%
Sn-Cl2	2.3656	2.4090	1.83%
O1-H1	0.9803	0.8200	16.3%
N1-N2	1.3563	1.371	1.08%
F1-C3	1.3384	1.360	1.61%
C5-F2	1.3557	1.360	0.31%
C5-C4	1.3872	1.371	1.16%

xyz coordinates of the optimized geometries using HSEH1PBE functional

Sn	1.960313	5.381978	5.153225
Cl	1.518408	3.356576	6.588149
Cl	1.418836	6.745826	7.171278
Cl	2.742911	3.779412	3.386555
Cl	4.371846	5.362417	6.039703
Cl	-0.261855	5.381152	4.341681
Cl	2.683917	7.333745	3.907815
F	6.523035	1.558048	10.584922
O	3.260131	3.808861	9.039252
H	2.830469	3.504626	8.212299
N	4.571382	1.997279	7.157952
F	3.486012	-1.094141	12.944765
N	5.365865	5.517215	9.438756
N	3.788255	0.944088	7.500221
N	5.029543	7.335366	8.370605
N	3.677026	1.344376	5.349575

C	4.211995	2.011113	10.331782
N	6.597805	5.976631	9.109105
C	5.237250	1.261030	10.895264
C	4.417727	6.324632	8.979023
H	3.351790	6.165965	9.042587
C	4.457623	3.182265	9.376129
C	5.221103	4.267636	10.174629
H	6.226799	3.954803	10.450099
H	4.645342	4.463290	11.080544
C	5.308746	2.773944	8.148170
H	6.178231	2.201969	8.472032
H	5.636192	3.670281	7.618926
C	5.028221	0.213048	11.769851
H	5.858473	-0.346315	12.181472
C	2.913260	1.675902	10.710749
H	2.096753	2.254120	10.295837
C	3.718047	-0.083125	12.098904
C	4.510130	2.248472	5.860587
H	5.043332	3.005731	5.284428
C	6.359518	7.076802	8.451467
H	7.124055	7.705888	8.024705
C	3.231404	0.580690	6.384009
H	2.511583	-0.215383	6.286428
C	2.651446	0.633264	11.587499
H	1.639578	0.374515	11.873845
H	4.528645	8.025196	7.708979
H	3.284515	1.438681	4.416198
O	3.687633	8.843074	6.697002
H	2.780032	8.524134	6.853829
H	3.824752	8.588690	5.771231
O	5.955920	3.855161	3.700695
H	6.025414	4.725151	4.106941
H	5.123334	3.922262	3.211736

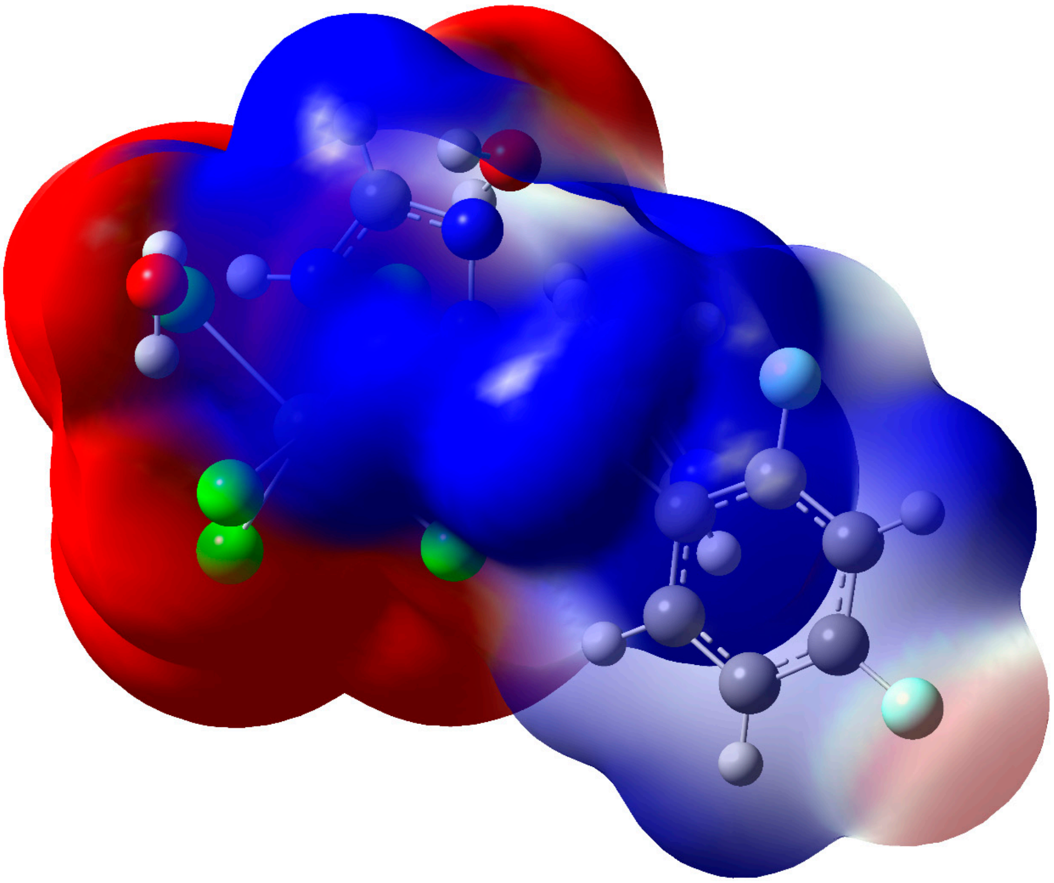

Figure S1: The electrostatic potential map of the (H2Fluconazole).$SnCl_6$.$2H_2O$

2.3.2. $SnCl_6$ internal modes

The Raman spectrum (Figure 6b) shows a weak band at 314 cm^{-1} is assigned to the (Sn-Cl) asymmetric stretching (ν_1(Sn-Cl)). The symmetric stretching (ν_2(Sn-Cl)) vibration and the bending vibration in the ν_5(Cl-Sn-Cl) plane are overlapped with other vibrations. The weak bands located at 171, 155 and 128 cm^{-1} in the Raman spectrum are attributed to the bending vibrations of (Cl-Sn-Cl) planes.

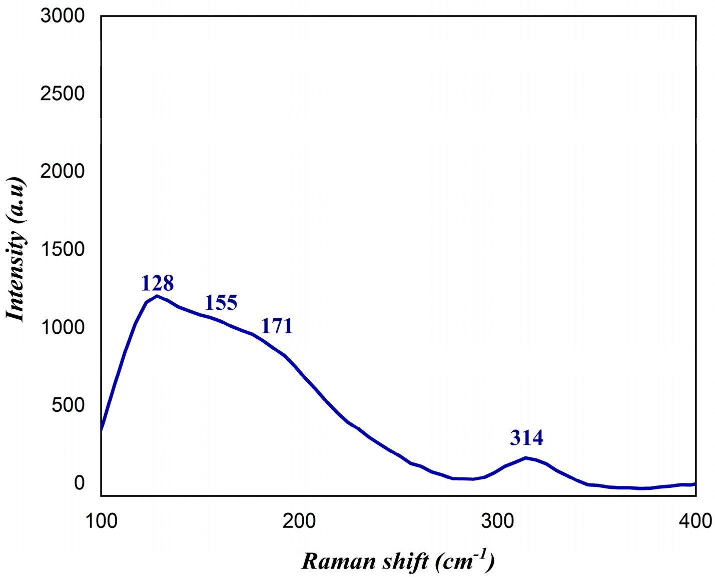

Figure S2. Raman spectrum of (H2Fluconazole).$SnCl_6.2H_2O$ at room temperature.
